# Knowledge and Attitude toward Human Papillomavirus Infection and Vaccination among Thai Women: A Nationwide Social Media Survey

**DOI:** 10.31557/APJCP.2020.21.10.2895

**Published:** 2020-10

**Authors:** Naratassapol Likitdee, Chumnan Kietpeerakool, Bandit Chumworathayi, Amornrat Temtanakitpaisan, Apiwat Aue-Aungkul, Wilasinee Nhokaew, Nampet Jampathong

**Affiliations:** *Department of Obstetrics and Gynaecology, Faculty of Medicine, Khon Kaen University, Khon Kaen, Thailand. *

**Keywords:** Human papillomavirus, vaccine- knowledge, attitude, nationwide, social media site

## Abstract

**Objectives:**

This study was performed first to assess Thai women’s knowledge and attitude toward Human papillomavirus (HPV) infection and vaccination and second to find out factors associated with knowledge in this regard.

**Methods:**

The survey announcement was advertised via Facebook from 17 May 2019 to 14 June 2019 to recruit women aged 18-26 years living in Thailand. A score below 5 out of total score of 10 on the survey was considered as a poor level of knowledge. Multivariate analysis was applied to identify factors associated with HPV infection and vaccination knowledge.

**Results:**

A total of 1,175 participants were recruited. The participants’ median age was 22 years. Approximately, 46% of the participants had poor level of knowledge regarding HPV infection and vaccination. Factors associated with poor knowledge included low educational level (adjusted OR, 1.35; 95% CI 1.04-1.77), low family income (adjusted OR, 2.14; 95% CI 1.65-2.78), being Christian (adjusted OR, 4.04; 95% CI 1.22-13.40), being engaged in sexual intercourse (adjusted OR, 0.75; 95%CI 0.58-0.97), and being unvaccinated against HPV infection (adjusted OR, 5.74; 95% CI 3.07-10.74).

**Conclusion:**

Nearly half of the Thai women who participated in the survey had poor level of knowledge regarding HPV infection and vaccination, indicating a need for more effective health education intervention. Factors associated with knowledge included socioeconomic status and sexual behavior.

## Introduction

Sexually acquired infection with high-risk human papillomavirus (HPV) is often a precursor to cervical cancer and pre-cancer (Burd, 2003; Saslow et al., 2012; Centers for Disease Control and Prevention, 2019a; World Health Organization, 2019a). Health education interventions to increase awareness regarding HPV prevention, including vaccination, are therefore necessary in order to reduce the rates of these cancers (Lowy et al., 2008; World Health Organization, 2014; Arrossi et al., 2017).

Various educational interventions have been implemented in order to increase HPV awareness and the rate of HPV vaccination, including distribution of information sheets and educational leaflets (either given directly to specific population or distributing among public people), forming social media campaigns, and designing health education websites (Hughes et al., 2009; Rosen et al., 2018; Leung et al., 2019). Previous studies have found that knowledge and attitudes toward HPV infection and vaccination are individual factors associated with the success of cervical cancer prevention programs, making them important issues to consider when designing a health education program (Nganwai et al., 2008; Gunasekaran et al., 2013; Kittisiam et al., 2016; Chen et al., 2018; He and He, 2018; Btoush et al., 2019).

As knowledge and attitudes toward HPV infection and vaccination have been found to vary by region (Remschmidt et al., 2014; Kittisiam et al., 2016; Chen et al., 2018; Btoush et al., 2019; Shabani et al., 2019), this study was conducted to assess these issues in Thai women using Facebook as a tool for nation-wide recruitment. Discovering factors associated with low levels of knowledge can provide foundational information for designing an effective cervical cancer prevention program for a specific region.

## Materials and Methods

*Study setting and design*

This cross-sectional study was conducted using an electronic survey design for assessing the respondents’ knowledge and attitudes toward HPV infection and vaccination. Participants were recruited via Facebook. The study was conducted based on the Checklist for Reporting Results of Internet E-Surveys (CHERRIES; Eysenbach, 2004) guidelines.

*Participants*

Thai women aged 18-26 years old were invited to participate in this study through targeted advertising on Facebook from 17 May 2019 to 14 June 2019. Those aged>26, were not willing to participate, provided incomplete questionnaires, or completed the questionnaire in less than 1 minute were excluded.

*Informed consent process*

We did the survey via Facebook. Eligible participants were asked to click on a link to read a consent form covering the purposes of the research, benefits, potential discomfort, duration of the survey (5-10 minutes), data protection and privacy, and incentives. The informed consent process was completed when the participants signed the informed consent form by providing their e-mail address and clicking “submit” button.

*Data protection*

Only one of the researchers (NL) and one programmer were authorized to access the e-survey database. The questionnaires were anonymous. Participants’ email addresses were extracted from the questionnaire and were deleted after the research was completed. Data confidentiality protocols were implemented in accordance with the Declaration of Helsinki.

*Development and pre-testing*

Our Thai-language questionnaire assessed the respondents’ knowledge and attitudes regarding HPV infection and vaccination. It was designed to be viewable on both mobile devices and personal computers. It was approved by five experts in gynecologic oncology using standard resources from an international organization (Centers for Disease Control and Prevention, 2019b; World Health Organization; 2019b). It had a content validity index (CVI) of 0.97, Kuder-Richardson Formula (KR-20) score of 0.66, and Cronbach’s alpha coefficient of 0.67. The system was tested, and the required time to complete the questionnaire was measured prior to implementation.

*Contact mode*

The researchers initially contacted eligible participants through an advertisement on Facebook. The electronic questionnaire was automatically sent out to participants by email after completing the informed consent process.

*Advertising the survey*

We choose Facebook as the platform on which to advertise the survey. An announcement was posted on our department’s official Facebook page (Department of Obstetrics and Gynaecology Faculty of Medicine Khon Kaen University). The paid advertisement was shown between 17 May 2019 and 14 June 2019. Specific targeted population was selected (e.g. females aged 18-26 years living in Thailand) via “Facebook Ads Manager”, The advertisement statistics were analyzed and reported by Facebook.

*Survey administration*

This was an open e-survey. Eligible participants were able to click on a link to direct them to the informed consent form. After completing the informed consent form, the participants would automatically receive a link to the electronic questionnaire via email.

*Context*

We posted the survey announcement on the “Department of Obstetrics and Gynaecology Faculty of Medicine Khon Kaen University” Facebook page, as the platform allows for the boosting of paid advertisements and because the page was related to the research topic.

*Mandatory/voluntary*

This was a voluntary survey. Even after giving informed consent, participants were able to discontinue the survey at any time if they felt uncomfortable.

*Incentives*

The researchers provided details about incentives in the consent form. Participants had an opportunity to win a monetary prize.

*Numbers of items and pages*

The questionnaire consisted of 3 pages covering (1) personal data, (2) knowledge regarding HPV infection and vaccination, and (3) attitude toward HPV infection and vaccination.

Page 1 consisted of 12 questions regarding personal data, including age, region of residence, occupation, education, monthly family income, religion, current sexual partner(s), age of first sexual intercourse, number of sexual partners, history of HPV vaccination, age at first HPV vaccination, and source of information about the HPV vaccine.

Page 2 consisted of 10 questions to assess the respondents’ knowledge regarding HPV infection and vaccination (response options: “yes,”“no,”“don’t know”). The results were then classified as either good or poor knowledge.

Page 3 consisted of 12 questions regarding the respondents’ attitude toward HPV infection and vaccination using a four-point Likert scale (“strongly agree,” “moderately agree,” “slightly agree,” and “disagree”).

*Completeness check and review step*

If a page of the questionnaire was incomplete, the participant was not allowed to continue to the next page. Participants were allowed to edit their previous answers by clicking the “back” button and resubmitting. Participants were not allowed to edit their answers after completing all 3 pages of the questionnaire.

*Registration*

The e-survey registration was designed to prevent duplicate entries from the same user by not allowing access from the same email address more than once.

*Log file analysis*

Time spent completing the questionnaire was measured and used to identify multiple entries. The timeframe that was used as a cut-off point was 1 minute. If a participant spent less time than the considered one for completing the questionnaire, she was excluded from the study.

*Sample size*

The minimum sample size needed for this study was 344 participants based on previous studies (Stridh and Hammer, 2014; Kittisiam et al., 2016).

*Statistical analysis*

A score below 5 on the HPV knowledge section of the questionnaire was considered as a poor level of knowledge. Low monthly family income was defined as a total monthly income below the national average (20,000 Baht or 660 USD per month).

Descriptive statistics, including numbers, percentages, and medians with interquartile ranges, were used. Statistical analysis was carried out using SPSS (version 17.0). Univariate analysis was carried out to identify factors potentially related to HPV infection and vaccination knowledge, including region of residence, occupation, education level, monthly family income, religion, sexual behavior, and vaccination against HPV. Variables with statistical significance in the univariate analysis (a 95% confidence interval of unadjusted odds ratio that did not include the unity) were then included in stepwise multivariate logistic regression analysis to determine which factors were independently associated with knowledge in this regard.

*Ethical consideration*

The study protocol and consent form were approved by the Ethics Committee of Human Research, Khon Kaen University (HE621188).

## Results

The survey announcement was viewed 237,513 times, and informed consent page had 1,909 visitors. A total of 1,682 visitors (88.11 %) signed the informed consent form, and 1,295 participants completed the questionnaire, resulting in a completion rate of 76.99%. One hundred twenty of these participants were excluded due to their age.

The demographic characteristics of the 1,175 participants are displayed in Table 1. The participants’ median age was 22 years. Approximately three-fourths of the participants were between 20 and 26 years old. Most of the participants resided in the northeast region of Thailand (37.4%), followed by the central region (22.6%), northern (16.9%), southern (12.3%), and Bangkok (10.8%). Most of the participants (91%) were Buddhists. More than half (51.1%) had a bachelor’s degree. Approximately, 42% had monthly family income more than the national average household income (20,000 Baht or 660 USD; National Statistical Office of Thailand, 2019a). Most of the participants (54%) had no current sexual partner.

Almost all participants (90.9%) were unvaccinated against HPV. Among those who were vaccinated, 98 were first vaccinated at age between 15 and 26 years old, and the remaining 9 were vaccinated before 15 years of age. Most of the participants heard about the HPV vaccine from social media/Internet (86.7%), followed by healthcare providers (44.7%), television (20.5%), friends (17.6%), relatives (10.1%), and newspapers (5.4%).

Table 2 shows the scores with regard to HPV infection and vaccination knowledge. Approximately, 77% of the participants knew that HPV infection was the main cause of cervical cancer, and 79% knew that HPV could be transmitted via sexual intercourse. However, only 46.7% knew that using condom did not provide 100% protection against HPV. Most participants mistakenly thought that HPV infection could be detected by Pap smear (94.3%), most HPV infections could heal by themselves (91.9%), and HPV infection usually had no specific symptoms or signs (88.0%). The mean ± SD HPV infection and vaccination knowledge was 4.5+ 2. Approximately, 46% of the participants had poor level of knowledge in this regard.

Table 3 shows the results of multinomial logistic regression analysis.Factors that were independently associated with a poor level of knowledge included education level lower than university (adjusted OR, 1.35; 95%CI 1.04-1.77), low monthly family income (adjusted OR, 2.14; 95%CI 1.65-2.78), being Christian (adjusted OR, 4.04; 95%CI 1.22-13.40), having a sexual partner (adjusted OR, 0.75; 95%CI 0.58-0.97), and being unvaccinated against HPV (adjusted OR, 5.74; 95%CI 3.07-10.74).

Attitudes towards HPV infection and vaccination are displayed in Table 4. Three-fourths of the participants (73.4%) believed that sexual activity was a risk factor for HPV infection, and most (97.3%) thought that people with multiple sexual partners were at higher risk of becoming infected with HPV. Nearly all participants agreed that HPV prevention was important for women (99.2%) and that HPV education should be implemented in schools (99.5%).

Approximately, 94% of the participants believed that HPV vaccine was safe and highly effective. Although the HPV vaccine cannot protect against all strains of HPV, almost all participants (98.4%) thought it was still necessary to be vaccinated against HPV. About 97% of the participants thought that it was better for both men and women to get vaccinated before becoming sexually active. Although the HPV vaccine is not included in the national universal health coverage scheme, the majority of the participants (95.9%) thought it was worth the price.

Nearly, 93% of the participants stated that they would be able to get the HPV vaccine without the permission of their parents or guardians. In terms of cervical cancer screening, nearly all participants (98.7%) agreed that women who were vaccinated against HPV still needed to undergo cervical cancer screening.

**Table 1:** Demographic Characteristics of the Participants

		n	(%)
Age (years), median (IQR)		22	(20-24)
Age (years)			
<20		275	(23.4)
20-26		900	(76.6)
Region of residency	Female population, 2017*		
Northeastern region	33.20%	440	(37.4)
Central region	25.70%	265	(22.6)
Northern region	18.30%	198	(16.9)
Southern region	14.20%	145	(12.3)
Bangkok	8.60%	127	(10.8)
Occupation			
Unemployed		799	(68)
Private employee		217	(18.5)
Government officer		159	(13.5)
Education			
No education		1	(0.1)
Primary school		3	(0.3)
High school/diploma		554	(47.1)
Bachelor’s degree		600	(51.1)
Master or doctor degree		17	(1.4)
Monthly family income (Baht)			
<20,000		687	(58.5)
>20,000		488	(41.5)
Religion			
Buddhist		1,069	(91)
Christian		35	(3)
Muslim		47	(4)
None		24	(2)
Has a sexual partner			
Yes		545	(46.4)
No		630	(53.6)
Age at first intercourse (years)			
<14		31	(2.6)
>14		514	(43.7)
Not yet		630	(53.6)
Number of sexual partners			
1		259	(22)
> 1		286	(24.4)
None		630	(53.6)
Vaccinated against HPV (at least one dose)			
Yes		107	(9.1)
No		1,068	(90.9)
Age at first dose of HPV vaccine (year)			
< 15		9	(0.8)
15 – 26		98	(8.3)
Not yet		1,068	(90.9)
		n	(%)
Source of information about the HPV vaccine			
Social media/internet		1,019	(86.7)
Television		241	(20.5)
Newspapers		63	(5.4)
Healthcare provider		525	(44.7)
Family/relatives		119	(10.1)
Friends		207	(17.6)

*Thai female population in the year 2017 (National Statistical Office of Thailand; 2019b); IQR, Interquartile Range; HPV, Human papillomavirus

**Table 2:** Knowledge of HPV Infection and Vaccination

Statement	TRUE		FALSE	
	n	(%)	n	(%)
1. HPV can be transmitted via sexual intercourse.	928	(79	247	(21)
2. Most people with HPV infections present with itching, abnormal vaginal discharge, or vaginal bleeding.	148	(12.6)	1,027	(87.4)
3. HPV infection can be treated with antibiotics.	400	(34)	775	(66)
4. Most HPV infections can be healed by themselves.	95	(8.1)	1,080	(91.9)
5. Using a condom can provide 100% protection against HPV.	549	(46.7)	626	(53.3)
6. The HPV vaccine can provide 100% protection against HPV.	586	(49.9)	589	(50.1)
7. The HPV vaccine is recommended for women aged 9-26 years.	802	(68.3)	373	(31.7)
8. HPV infection is the main cause of cervical cancer.	904	(76.9)	271	(23.1)
9. The HPV infection can be detected by conventional cervical cancer screening methods or Pap smear.	67	(5.7)	1,108	(94.3)
10. Women who have been vaccinated against HPV do not need to perform cervical cancer screening.	866	(73.7)	309	(26.3)

*HPV, Human papillomavirus

**Table 3:** Factors Associated with a Poor Level of Knowledge Regarding HPV Infection and Vaccination

Variables	Total		Poor (n=537)		Good (n=638)		OR (95% CI)	aOR (95% CI)
	n	(%)	n	(%)	n	(%)		
Region of residency								
Bangkok	127	10.8	45	8.4	82	12.9	1	1
Northeastern region	440	37.5	216	40.2	224	35.1	1.76 (1.17-2.64)	1.21 (0.77-1.88)
Central region	265	22.6	117	21.8	148	23.2	1.44 (0.93-2.23)	NA
Northern region	198	16.9	92	17.1	106	16.6	1.58 (1.00-2.50)	NA
Southern region	145	12.3	67	12.5	78	12.2	1.57 (0.96-2.55)	NA
Occupation								
Employed	376	32	150	27.9	226	35.4	1	1
Unemployed	799	68	387	72.1	412	64.6	1.42 (1.10-1.81)	1.21 (0.90-1.62)
Education								
University degree	617	52.5	249	46.4	368	57.7	1	1
Less than university	558	47.5	288	53.6	270	42.3	1.58 (1.25-1.99)	1.35 (1.04-1.77)
Monthly family income (Baht)								
>20,000	488	41.5	157	29.2	331	51.9	1	1
<20,000	687	58.5	380	70.8	307	48.1	2.61 (2.05-3.32)	2.14 (1.65-2.78)
Religion								
None	24	2	6	1.1	18	2.8	1	1
Buddhist	1,069	91	486	90.5	583	91.4	2.5 (0.99-6.35)	NA
Christian	35	3	22	4.1	13	2	5.08 (1.61-16.04)	4.04 (1.22-13.40)
Muslim	47	4	23	4.3	24	3.8	2.87 (0.97-8.52	NA
Sexually active								
Yes	545	46.4	269	50.1	276	43.3	1	1
No	630	53.6	268	49.9	362	56.7	0.76 (0.60-0.96)	0.75 (0.58-0.97)
Age at first intercourse (years)								
Not yet	630	53.6	268	49.9	362	56.7	1	NA
<14	31	2.6	17	3.2	14	2.2	1.64 (0.79-3.39)	NA
>14	514	43.7	252	46.9	262	41.1	1.3 (1.03-1.64)	NA
Number of sexual partners								
None	630	53.6	630	53.6	362	56.7	1	NA
1	259	22	129	24	130	20.4	1.34 (1.00-1.79)	NA
>1	286	24.3	140	26.1	146	22.9	1.3 (0.98-1.71)	NA
Vaccinated against HPV								
Yes	107	9.1	12	2.2	95	14.9	1	1
No	1,068	90.9	525	97.8	543	85.1	7.65 (4.15-14.12)	5.74 (3.07-10.74)

*NA, not assessed; HPV, Human papillomavirus

**Table 4:** Attitude towards HPV Infection and Vaccination

Statement	Strongly agree n (%)		Moderately agree n (%)		Slightly agree n (%)		Disagree n (%)	
1. I think I am at risk of HPV infection if I am sexually active.	440	37.4	423	36	218	18.6	94	8
2. Preventing HPV infection is important for women.	1,110	94.5	55	4.7	5	0.4	5	0.4
3. People who have multiple sexual partners have higher risk of becoming infected with HPV.	1,006	85.6	137	11.7	26	2.2	6	0.5
4. Schools should implement HPV education.	1,100	93.6	69	5.9	6	0.5	0	0
5. I think the HPV vaccine is worth the price.	800	68.1	327	27.8	47	4	1	0.1
6. It is better to be vaccinated against HPV before becoming sexually active.	989	84.2	146	12.4	33	2.8	7	0.6
7. It is preferable that both men and women be vaccinated against HPV.	986	83.9	146	12.4	33	2.8	10	0.9
8. I am sure that the HPV vaccine is highly effective.	480	40.9	616	52.4	73	6.2	6	0.5
9. Although the HPV vaccine cannot protect against all strains of HPV, I think it is necessary.	954	81.2	202	17.2	19	1.6	0	0
10. I am sure that the HPV vaccine is safe.	595	50.6	515	43.8	64	5.5	1	0.1
11. Women who have been vaccinated against HPV still need to undergo cervical cancer screening.	1,039	88.4	121	10.3	14	1.2	1	0.1
12. I can get the HPV vaccine without asking for permission from my parents/guardians.	853	72.6	236	20.1	67	5.7	19	1.6

* HPV, Human papillomavirus

## Discussion

About 46% of the Thai women who participated in this study had poor level of knowledge regarding HPV infection and vaccination, particularly with regard to transmission routes, symptoms, and prevention of HPV infection. The factors affecting HPV knowledge were education level, family income, having sexual partners, religion, and vaccination status.

The level of knowledge regarding HPV infection and vaccination was varied by region. Our study showed that approximately 54% of Thai women had good level of HPV knowledge. Previous studies found that over 90% and around 63% of participants in Germany and Australia, respectively, were aware of HPV (Gunasekaran et al., 2013; Remschmidt et al., 2014). However, a study in South America found that only 55% of mothers had ever heard about HPV (Btoush et al., 2019). Two studies in China found that approximately 45% of women in Mainland China had heard about HPV, but only 29% of those in western China had this knowledge (Chen et al., 2018; He and He, 2018). Another study in South Africa found that 29% of participants in urban areas and 27% in rural areas knew about HPV (Shabani et al., 2019). A large study conducted in Thailand found that nearly 66% of their participants knew about HPV (Kittisiam et al., 2016).

Most participants in our study had some misunderstandings about HPV. Only 12% knew that HPV infection usually had no signs and symptoms, and nearly 54% thought that condom could fully protect them against HPV infection. Our findings were consistent with the results of studies conducted in China and the Eastern United States, which found that the majority of participants lacked knowledge about HPV symptoms and prevention (He and He, 2018; Btoush et al., 2019). Another study in Germany found that almost half of the participants did not know that condom use alone did not sufficiently protect them against HPV infection (Remschmidt et al., 2014).

However, some aspects of HPV infection seemed to be well understood by the majority of participants in our study. For example, 77% knew that HPV infection was the main cause of cervical cancer, which was higher than the results reported in a previous study conducted in China (53.4%; He and He, 2018), but lower than the results reported in a study in Australia (92.4%; Gunasekaran et al., 2013). Moreover, approximately 79% of the participants in our study knew that HPV could be transmitted via sexual intercourse, which was consistent with the results of previous studies in the United States and Australia, revealing that 85% and 73.4% of their participants, respectively, knew that HPV infection was a sexually transmitted disease (Gunasekaran et al., 2013; Kasymova et al., 2019).

Our study found that low education level, low monthly family income, and having a sexual partner were significantly associated with low HPV knowledge. Previous studies reported that education level, socio-economic status, and employment status were positively associated with percentage of correct answers on similar surveys (Gunasekaran et al., 2013; Kittisiam et al., 2016; Chen et al., 2018; He and He, 2018).

 A recent study noted an association between place of residence and level of HPV knowledge (Btoush et al., 2019). However, our results failed to confirm this finding. In our study, place of residence was associated with HPV knowledge based on univariate analysis results, but was not statistically significant after adjustment for other variables.

We anticipated that women who were vaccinated would have a higher level of HPV knowledge compared to those who were not. This anticipation was due to the fact that those who chose to be vaccinated had often done prior research about HPV or had received information from their healthcare providers. In fact, unvaccinated participants in our study were approximately six times as likely to have poor knowledge when compared to vaccinated participants. Interestingly, our study noted an association between being Christian and possessing a low level of HPV knowledge. However, a larger scale study should be conducted to confirm this finding.

Facebook has been shown to be an effective tool for enrolling participants in research (Kapp et al., 2013; Kayrouz et al., 2016; Akers and Gordon, 2018). We found two previous studies that used Facebook to collect data regarding HPV knowledge among young women. In those studies, the demographic characteristics of the participants did not differ from the distributions specified by the National Bureau of Statistics, indicating that Facebook is an appropriate tool for a nation-wide study (Gunasekaran et al., 2013; Remschmidt et al., 2014). We also found that the geographic region of the participants was nearly the same as the data from the National Statistical Office of Thailand.

This was the first study that focused on knowledge and attitudes regarding HPV using a nationwide social media survey in Thailand. Our study was able to recruit a large sample size covering all of the different regions of Thailand, thus representing nationwide results. However, the fundamental limitation of this study was that its results cannot be validated as they were based on self-reported data.

Our results indicated that women who had a low educational background, low socioeconomic status, and those who were unvaccinated against HPV were more likely to have a low level of HPV knowledge. These groups of women should; therefore, be targeted to improve the effectiveness of cervical cancer prevention strategies.

In conclusion, nearly half of the Thai women in our study had poor level of knowledge regarding HPV infection and vaccination. In addition, they had misconceptions about HPV. Educational interventions seem necessary to improve the understanding of this population.
